# Supplementary material for: The AM-4 Family of Layered Titanosilicates: Single-Crystal-to-Single-Crystal Transformation, Synthesis and Ionic Conductivity
Source: Materials (Basel). 2023 Dec 25;17(1):111. doi: 10.3390/ma17010111 (PMC10780191; doi:10.3390/ma17010111)
Supplement: Supplementary file 1 [file materials-17-00111-s001.zip › materials-2593548-supplementary.pdf]

Supplementary Materials: Properties of compounds AM-4 family: single to single crystal transformation, synthesis, ion-conductivity

Galina O. Kalashnikova [1], Sergey V. Krivovichev [1,2], Victor N. Yakovenchuk[1,3], Ekaterina A. Selivanova [1,3], Margarita S. Avdontseva [2], Gregory Yu. Ivanyuk [1], Yakov A. Pakhomovsky [1,3], Darya V. Gryaznova [1], Natalya A. Kabanova [4,5], Yelizaveta A. Morkhova [5,6], Olga Yu. Sinel'shikova [7], Vladimir N. Bocharov [8], Anatoly N. Nikolaev [1,9], Olga F. Goychuk[1,4] and Taras L. Panikorovskii [4]

1. X-Ray Supplementary Data

Table S1. Fractional atomic coordinates and equivalent isotropic displacement parameters (Å^2) for lintisite sample.

Site	occupancy	*x/a*	*y/b*	*z/c*	U^{ani}
Ti1	Ti	0.33495(2)	0.09521(4)	0.37533(6)	0.00623(10)
Si2	Si	0.26371(2)	0.40758(6)	0.29046(10)	0.00678(12)
Si1	Si	0.40747(2)	0.40135(6)	0.46088(10)	0.00806(12)
Na1	Na	0.33483(3)	0.31265(10)	0.88442(16)	0.01664(19)
Na2	Na	½	0.15123(18)	¼	0.0271(3)
O6	O	0.20710(5)	0.41625(15)	0.2879(3)	0.0083(3)
O7	O	0.36388(5)	0.07017(15)	0.0716(3)	0.0095(3)
O5	O	0.28632(5)	0.23888(15)	0.2577(3)	0.0104(3)
O4	O	0.28461(4)	0.48802(16)	0.5531(2)	0.0093(3)
O3	O	0.37494(5)	0.24912(16)	0.5101(3)	0.0112(3)
O2	O	0.38970(5)	0.47326(17)	0.1815(3)	0.0124(3)
O1	O	0.46223(5)	0.37352(18)	0.4809(3)	0.0136(3)
O8	O	0.45502(7)	0.1035(2)	-0.1166(4)	0.0290(4)
Li1	Li	½	0.5111(7)	¼	0.0179(10)
H8A	H	0.4265(15)	0.097(4)	-0.075(8)	0.049(11)
H8B	H	0.4552(14)	0.171(5)	-0.239(9)	0.054(11)

Table S2. Fractional atomic coordinates and equivalent isotropic displacement parameters (Å^2) for kukisvumite sample.

Site	occupancy	*x/a*	*y/b*	*z/c*	U^{ani}
Ti1	Ti	0.41640(2)	0.34498(5)	0.36755(9)	0.00612(12)
Zn1	$Zn_{0.50}$	¼	¾	0.2089(2)	0.0141(2)
Si2	Si	0.34371(3)	0.64912(9)	0.44830(15)	0.00743(16)
Si1	Si	0.48623(3)	0.65771(8)	0.28857(15)	0.00592(15)
Na2	Na	0.41567(5)	0.56145(15)	0.8766(3)	0.0164(3)
O8	O	0.38796(7)	0.3194(2)	0.0614(4)	0.0088(4)
O1	O	0.54214(7)	0.6666(2)	0.2915(4)	0.0078(4)
O3	O	0.46426(7)	0.4881(2)	0.2539(4)	0.0101(4)
O4	O	0.37719(7)	0.5002(2)	0.4945(4)	0.0111(4)
O2	O	0.46546(6)	0.7384(2)	0.5490(4)	0.0086(4)
O6	O	0.35966(8)	0.7260(3)	0.1732(5)	0.0173(5)
O5	O	0.29033(8)	0.6103(3)	0.4588(7)	0.0319(7)
Na1	$Na_{0.50}$	0.24970(13)	0.4092(5)	0.2112(7)	0.0304(11)
O7B	$O_{0.50}$	0.20734(19)	0.3515(7)	0.5689(12)	0.0263(12)
O7A	$O_{0.50}$	0.20313(19)	0.3530(7)	0.3697(12)	0.0275(12)

Table S3. Fractional atomic coordinates and equivalent isotropic displacement parameters (Å^2) for K3 sample.

Site	occupancy	*x*/*a*	*y*/*b*	*z*/c	U^{ani}
Ti1	Ti	0.5950(2)	0.6531(7)	-0.0923(13)	0.016(4)
Ti2	Ti	0.5949(3)	-0.1286(7)	0.4069(12)	0.016(4)
Si2	Si	0.5205(4)	0.1765(11)	0.276(2)	0.016(4)
Si1	Si	0.5213(4)	0.3463(11)	-0.2263(18)	0.014(4)
Si4	Si	0.6839(4)	0.3557(13)	1.030(2)	0.021(4)
Si3	Si	0.6845(4)	0.1574(12)	0.5453(18)	0.019(4)
O4	O	0.4492(10)	0.201(3)	0.266(5)	0.023(7)
O13	O	0.6372(9)	0.697(3)	-0.382(5)	0.020(5)
O10	O	0.7492(8)	0.414(3)	1.042(6)	0.020(5)
O2	O	0.4510(8)	0.330(2)	-0.220(5)	0.011(6)
O6	O	0.5466(11)	0.267(2)	0.522(5)	0.015(6)
O1	O	0.5422(9)	0.521(2)	-0.211(5)	0.013(6)
O9	O	0.6447(16)	0.503(5)	1.046(9)	0.063(11)
O5	O	0.5421(11)	0.005(3)	0.275(6)	0.032(7)
O7	O	0.6435(9)	0.009(3)	0.511(5)	0.020(6)
O3	O	0.5450(13)	0.259(3)	0.017(5)	0.024(7)
O12	O	0.6386(10)	-0.181(3)	0.078(6)	0.028(7)
O11	O	0.676(2)	0.263(5)	1.314(10)	0.079(15)
O8	O	0.6718(13)	0.244(3)	0.805(6)	0.036(8)
O14	O	0.7521(14)	0.097(4)	0.501(10)	0.061(12)

Table S4. Fractional atomic coordinates and equivalent isotropic displacement parameters (Å^2) for L3 sample.

Site	occupancy	*x*/*a*	*y*/*b*	*z*/c	U^{ani}
Ti1	Ti	0.3101(2)	0.6407(2)	0.5104(5)	0.0197(11)
Si2	Si	0.4579(3)	0.3341(4)	0.7085(7)	0.0184(12)
Si1	Si	0.1314(4)	0.3508(4)	0.3001(8)	0.0250(13)
O5	O	0.5967(7)	0.3174(10)	0.7657(16)	0.0185(19)
O4	O	0.4179(8)	0.5097(9)	0.6826(17)	0.022(2)
O7	O	0.2247(8)	0.6899(11)	0.7863(18)	0.025(2)
O6	O	0.4082(7)	0.2431(10)	0.4434(16)	0.020(2)
O3	O	0.2119(8)	0.4961(12)	0.352(2)	0.032(2)
O1	O	0.0001(9)	0.4081(13)	0.247(2)	0.041(3)
O2	O	0.1512(11)	0.2632(15)	0.048(2)	0.051(3)

Table S5. Selected interatomic distances for lintisite.

Atom	Atom	Length/Å	Atom	Atom	Length/Å
Ti1	O6^{4}	2.1438(14)	Na1	O5^{9}	2.4875(16)
Ti1	O6^{5}	2.1190(13)	Na1	O4	2.6859(16)
Ti1	O7^{1}	1.9274(14)	Na1	O4^{7}	2.4083(16)
Ti1	O7	1.8094(14)	Na1	O3	2.3418(16)
Ti1	O5	1.9479(14)	Na1	O2^{7}	2.6469(17)
Ti1	O3	1.8748(14)	Na1	O2^{9}	2.5816(17)
Si1	O3	1.6264(14)	Na2	O1	2.5094(19)
Si1	O2	1.6523(15)	Na2	O1^{12}	2.5094(19)
Si1	O2^{7}	1.6604(15)	Na2	O8^{11}	2.636(2)
Si1	O1	1.5821(15)	Na2	O8^{1}	2.636(2)

Atom	Atom	Length/Å	Atom	Atom	Length/Å
			Na2	O8	2.318(2)
Si2	O6	1.6166(14)	Na2	O8[12]	2.318(2)
Si2	O5	1.5966(14)			
Si2	O4	1.6359(14)	Li1[8]	O1	2.014(3)
Si2	O4[6]	1.6469(14)	Li1	O1	2.012(4)
			Li1[8]	O1	2.014(3)
Na1	O6[4]	2.4629(16)	Li1	O1	2.012(4)
Na1	O7[9]	2.4387(16)			

[1]+X,-Y,1/2+Z; [2]+X,-Y,-1/2+Z; [3]+X,+Y,-1+Z; [4]1/2-X,1/2-Y,1-Z; [5]1/2-X,-1/2+Y,1/2-Z; [6]+X,1-Y,-1/2+Z; [7]+X,1-Y,1/2+Z; [8]1-X,1-Y,1-Z; [9]+X,+Y,1+Z; [10]1-X,-Y,1-Z; [11]1-X,-Y,-Z; [12]1-X,+Y,1/2-Z; [13]1-X,1-Y,-Z.

Table S6. Selected interatomic distances for kukisvumite.

Atom	Atom	Length/Å	Atom	Atom	Length/Å
Ti1	O8[2]	1.920(2)	Na2	O8[10]	2.427(2)
Ti1	O8	1.807(2)	Na2	O1[4]	2.468(2)
Ti1	O1[4]	2.144(2)	Na2	O3[10]	2.496(2)
Ti1	O1[5]	2.113(2)	Na2	O4	2.340(3)
Ti1	O3	1.943(2)	Na2	O2	2.700(2)
Ti1	O4	1.870(2)	Na2	O2[7]	2.414(2)
			Na2	O6[7]	2.659(3)
Zn1	O5[6]	2.120(3)	Na2	O6[10]	2.646(3)
Zn1	O5[8]	2.120(3)			
Zn1	O5	2.120(3)	Na1[11]	O5	2.459(5)
Zn1	O5[9]	2.120(3)	Na1	O5	2.454(5)
			Na1	O7B[8]	1.528(7)
Si1	O1	1.617(2)	Na1	O7B	2.284(7)
Si1	O3	1.599(2)	Na1	O7B[1]	2.657(7)
Si1	O2	1.637(2)	Na1	O7A[12]	2.758(7)
Si1	O2[6]	1.647(2)	Na1	O7A[8]	2.292(7)
			Na1	O7A	1.651(6)
Si2	O4	1.622(2)			
Si2	O6	1.644(2)			
Si2	O6[7]	1.653(2)			
Si2	O5	1.579(3)			

[1]+X,1/2-Y,-1/2+Z; [2]+X,1/2-Y,1/2+Z; [3]+X,+Y,-1+Z; [4]1-X,1-Y,1-Z; [5]1-X,-1/2+Y,1/2-Z; [6]+X,3/2-Y,-1/2+Z; [7]+X,3/2-Y,1/2+Z; [8]1/2-X,+Y,-1/2+Z; [9]1/2-X,3/2-Y,+Z; [10]+X,+Y,1+Z; [11]1/2-X,+Y,1/2+Z; [12]1/2-X,1/2-Y,+Z.

Table S7. Selected interatomic distances for K3.

Atom	Atom	Length/Å	Atom	Atom	Length/Å
Ti1	O4[3]	2.03(3)	Si1	O3	1.58(3)
Ti1	O13	1.84(3)			
Ti1	O2[4]	2.12(2)	Si2	O4	1.68(2)
Ti1	O1	1.80(2)	Si2	O6	1.62(3)
Ti1	O9[5]	1.90(4)	Si2	O5	1.58(3)
Ti1	O12[1]	1.98(3)	Si2	O3	1.63(3)

Ti2	O4[6]	2.02(3)	Si3	O7	1.62(2)
Ti2	O13[7]	2.13(3)	Si3	O11[5]	1.53(5)
Ti2	O2[6]	1.99(2)	Si3	O8	1.58(3)
Ti2	O5	1.83(3)	Si3	O14	1.68(3)
Ti2	O7	1.74(2)			
Ti2	O12	2.05(3)	Si4	O10	1.61(2)
			Si4	O9	1.58(4)
Si1	O2	1.65(2)	Si4	O11	1.69(5)
Si1	O6[5]	1.60(3)	Si4	O8	1.55(3)
Si1	O1	1.60(2)			

[1]+X,1+Y,+Z; [2]+X,1+Y,-1+Z; [3]1-X,1/2+Y,1/2-Z; [4]1-X,1/2+Y,-1/2-Z; [5]+X,+Y,-1+Z; [6]1-X,-1/2+Y,1/2-Z; [7]+X,-1+Y,1+Z.

Table S8. Selected interatomic distances for L3.

Atom	Atom	Length/Å	Atom	Atom	Length/Å
Ti1	O5[3]	2.007(9)	Si1	O1	1.619(11)
Ti1	O5[4]	2.123(9)	Si1	O2	1.574(11)
Ti1	O4	1.828(9)	Si1	O2[5]	1.613(11)
Ti1	O7[1]	2.038(10)			
Ti1	O7	1.956(10)	Si2	O5	1.634(9)
Ti1	O3	1.814(10)	Si2	O4	1.607(9)
			Si2	O6[5]	1.603(9)
Si1	O3	1.586(10)	Si2	O6	1.608(9)

[1]+X,3/2-Y,-1/2+Z; [2]+X,3/2-Y,1/2+Z; [3]1-X,1-Y,1-Z; [4]1-X,1/2+Y,3/2-Z; [5]+X,1/2-Y,1/2+Z.

Table S9. Anisotropic Displacement Parameters (Å^2×10^3) for lintisite sample. The Anisotropic displacement factor exponent takes the form: $-2\pi^2[h^2a^{*2}U_{11}+2hka^*b^*U_{12}+\ldots]$.

Atom	U^{11}	U^{22}	U^{33}	U^{23}	U^{13}	U^{12}
Ti1	8.41(16)	5.06(16)	5.23(16)	0.04(10)	0.09(11)	-0.21(10)
Si2	9.3(2)	5.8(2)	5.2(2)	-0.12(17)	0.10(18)	0.62(16)
Si1	10.0(2)	7.5(2)	6.7(2)	0.18(17)	-0.46(18)	-0.44(16)
Na1	22.9(4)	13.0(4)	14.2(4)	-1.0(3)	5.5(3)	0.7(3)
Na2	28.2(7)	29.5(8)	23.4(7)	0	-4.4(6)	0
O6	8.0(6)	8.4(6)	8.4(6)	0.7(5)	-1.0(5)	0.0(4)
O7	11.9(6)	8.9(6)	7.6(6)	-0.6(5)	0.0(5)	-1.0(5)
O5	14.5(6)	8.0(6)	8.7(6)	-0.5(5)	-0.4(5)	2.1(5)
O4	11.0(6)	9.5(6)	7.5(6)	-2.4(5)	0.0(5)	0.3(4)
O3	14.2(6)	9.4(6)	10.1(6)	-0.5(5)	-0.7(5)	-2.7(5)
O2	14.9(7)	12.6(7)	9.7(7)	2.9(5)	-0.9(5)	-1.2(5)
O1	11.4(6)	15.9(7)	13.6(7)	-1.2(6)	-0.3(5)	0.9(5)
O8	17.6(9)	41.4(12)	27.9(10)	2.9(9)	-0.7(7)	-0.4(7)
Li1	18(2)	24(3)	12(2)	0	-0.6(19)	0

Table S10. Anisotropic Displacement Parameters (Å^2×10^3) for kukisvumite sample. The Anisotropic displacement factor exponent takes the form: -2π^2[h^{2}a*^{2}U$_{11}$+2hka*b*U$_{12}$+…].

Atom	U^{11}	U^{22}	U^{33}	U^{23}	U^{13}	U^{12}
Ti1	7.8(2)	4.7(2)	5.9(2)	0.26(15)	-0.36(16)	0.03(15)
Zn1	11.8(4)	13.5(4)	17.0(5)	0	0	0.0(3)
Si2	8.5(3)	5.9(3)	7.9(3)	0.3(2)	0.4(3)	0.3(2)
Si1	7.5(3)	5.0(3)	5.2(3)	0.2(2)	-0.6(2)	-0.7(2)
Na2	22.2(6)	11.9(6)	15.0(6)	-0.2(5)	-6.2(5)	-1.6(5)
O8	10.4(9)	7.6(8)	8.6(9)	-1.6(7)	-0.4(7)	1.4(7)
O1	7.0(8)	8.8(8)	7.7(9)	0.9(7)	-1.1(7)	0.0(6)
O3	13.5(9)	7.5(8)	9.3(9)	0.2(7)	0.6(8)	-3.1(7)
O4	12.5(9)	8.5(9)	12.2(10)	-1.2(7)	-0.3(8)	3.6(7)
O2	9.7(8)	9.3(8)	6.7(9)	-1.8(7)	-0.4(7)	0.4(7)
O6	26.0(11)	13.1(10)	12.8(11)	4.9(8)	1.5(9)	-0.1(8)
O5	8.3(10)	13.6(11)	74(2)	2.5(13)	2.3(12)	-1.1(8)
Na1	28.0(17)	44(2)	19.7(17)	0.3(14)	6.2(14)	-1.9(15)
O7B	18(2)	35(3)	26(3)	-8(2)	3(2)	0(2)
O7A	18(2)	38(3)	27(3)	11(2)	-1(2)	-2(2)

Table S11. Anisotropic Displacement Parameters (Å^2×10^3) for K3 sample. The Anisotropic displacement factor exponent takes the form: -2π^2[h^{2}a*^{2}U$_{11}$+2hka*b*U$_{12}$+…].

Atom	U^{11}	U^{22}	U^{33}	U^{23}	U^{13}	U^{12}
Ti1	26(5)	11(4)	12(5)	-1(2)	1(2)	1.2(19)
Ti2	35(5)	9(4)	5(4)	7(2)	-1(2)	1(2)
Si2	26(5)	10(5)	13(5)	3(3)	0(3)	3(3)
Si1	22(5)	13(5)	6(5)	4(3)	0(3)	-2(3)
Si4	31(6)	24(6)	10(5)	1(4)	0(4)	2(3)
Si3	35(6)	18(6)	4(5)	0(4)	-4(3)	-3(3)
O4	27(8)	21(8)	22(9)	0(6)	1(6)	-1(6)
O13	20(5)	20(5)	20(5)	0.1(9)	-0.1(9)	0.1(9)
O10	20(5)	20(5)	20(5)	0.1(9)	-0.1(9)	0.1(9)
O2	14(6)	12(7)	9(7)	0(4)	1(4)	-1(4)
O6	17(8)	14(8)	13(8)	2(6)	-2(5)	1(5)
O1	20(7)	10(8)	10(8)	-2(6)	-2(5)	-3(5)
O9	64(12)	62(12)	63(12)	1(7)	0(6)	1(6)
O5	34(9)	33(9)	29(9)	1(6)	0(6)	1(6)
O7	22(8)	19(8)	18(8)	-2(6)	-2(6)	-4(5)
O3	26(8)	23(8)	21(9)	2(6)	-7(6)	1(5)
O12	30(8)	27(9)	27(9)	0(6)	-2(6)	-2(6)
O11	79(15)	79(16)	78(16)	0(7)	1(7)	1(6)
O8	41(10)	35(10)	31(10)	0(6)	0(6)	-2(6)
O14	61(12)	61(12)	61(12)	-0.2(14)	-0.1(14)	0.0(14)

Table S12. Anisotropic Displacement Parameters (Å^2×10^3) for L3 sample. The Anisotropic displacement factor exponent takes the form: $-2\pi^2[h^2a^{*2}U_{11}+2hka^*b^*U_{12}+\ldots]$.

**Atom**	***U***^{11}	***U***^{22}	***U***^{33}	***U***^{23}	***U***^{13}	***U***^{12}
Ti1	37.7(17)	12.0(16)	10.5(16)	0.0(7)	7.4(11)	-0.7(7)
Si2	40(2)	12(2)	5.4(18)	-0.4(12)	8.0(14)	1.2(12)
Si1	46(3)	15(2)	15(2)	-2.3(12)	8.9(18)	-2.9(13)
O5	36(4)	13(4)	7(3)	-2(3)	6(3)	-1(3)
O4	45(5)	10(4)	11(4)	-2(3)	11(4)	2(3)
O7	40(5)	24(5)	13(4)	-1(4)	7(4)	1(4)
O6	36(4)	13(4)	12(4)	1(3)	5(3)	2(3)
O3	41(5)	23(5)	33(6)	-6(4)	10(4)	-7(4)
O1	55(7)	28(7)	42(8)	8(6)	14(6)	-1(5)
O2	87(9)	42(7)	26(6)	-14(5)	19(6)	-7(6)

Table S13. X-ray Rietveld refinement of the $Ti(Si_2O_5(OH))(OH)$ structure.

Chemical formula	$Ti(Si_2O_5(OH))(OH)$
M_r	432.09
Temperature (°C)	23(2)
Crystal system, space group	Monoclinic, $P2_1/c$
a, *b*, *c* (Å)	11.92376(45), 8.72691(57), 5.20727(37)
β (°)	100.8989(94)
V (Å^3)	532.08(11)
Z	12
D_x (Mg m^{-3})	2.70
Radiation type	Cu Kα
Data collection	
Diffractometer	Rigaku SmartLab SE
θ-range	1.5–120, Step size (°) 0.02
$(\sin \theta/\lambda)_{max}$ (Å^{-1})	0.562
Refinement	
	R_{wp} 0.064
	R_p 0.066
	R_{Bragg} 0.079
S	1.0
No. of parameters	53

Table S14. Li^+-, Zn^{2+}- Na^+-ionic conductivities in lintisite, kukisvumite and AM-4 from KMC modeling, respectively.

**Structure**	**σ (S/cm) at 300 K**	**σ (S/cm) at 400 K**	**σ (S/cm) at 500 K**	**σ (S/cm) at 600 K**	**σ (S/cm) at 700 K**	**σ (S/cm) at 800 K**
Lintisite	6.16×10^{-7}	8.28×10^{-7}	3.09×10^{-5}	1.02×10^{-4}	1.40×10^{-4}	3.61×10^{-4}
Kukisvumite	2.20×10^{-4}	1.09×10^{-3}	1.66×10^{-2}	1.94×10^{-1}	6.99×10^{-1}	2.51×10^{0}
AM-4	1.36×10^{-7}	2.13×10^{-6}	6.75×10^{-6}	1.73×10^{-5}	4.70×10^{-5}	6.28×10^{-4}

Table 15. H^+-ionic conductivities in lintisite, kukisvumite and AM-4 from KMC modeling, respectively.

Structure	σ (S/cm) at 300 K	σ (S/cm) at 400 K	σ (S/cm) at 500 K	σ (S/cm) at 600 K	σ (S/cm) at 700 K	σ (S/cm) at 800 K
Lintisite	3.34×10^{-5}	2.92×10^{-4}	8.24×10^{-3}	1.22×10^{-2}	2.53×10^{-2}	5.02×10^{-2}
Kukisvumite	1.75×10^{-5}	2.49×10^{-5}	2.53×10^{-2}	3.18×10^{-1}	4.21×10^{-1}	3.37×10^{-1}
AM-4	9.15×10^{-2}	3.08×10^{-1}	2.25×10^{0}	8.84×10^{0}	1.46×10^{0}	2.28×10^{0}

Table S16. Parameters obtained by the VASP optimization for model structure AM-4 and its derivatives after exchange Na-Li.

Parameters		Model AM-4		Model AM-4-I		model AM-4-II	
		$Na_6Ti_4Si_8O_{28}$		$Li_2Na_4Ti_4Si_8O_{28}$		$Li_4Na_2Ti_4Si_8O_{28}$	
		Initial	After optimisation	Initial	After optimisation	Initial	After optimisation
Parameters of elementary cell	***a***, Å	15.26	15.69	15.69	15.08	15.69	15.30
	b, Å	15.26	15.69	15.69	15.08	15.69	15.30
	c, Å	5.20	5.31	5.31	5.31	5.31	5.19
	α	89.29	88.61	88.61	91.95	88.61	86.07
	β	89.29	88.61	88.61	91.95	88.61	86.07
	γ	32.62	32.41	32.41	33.79	32.41	32.88
	**V**, $Å^3$	653.22	700.77	700.77	671.44	700.77	658.64
**Energy after optimisation, eV**		-339.54 eV		-342.19 eV		-343.18 eV	
